# Environmental Viral Metagenomics Analyses in Aquaculture: Applications in Epidemiology and Disease Control

**DOI:** 10.3389/fmicb.2016.01986

**Published:** 2016-12-14

**Authors:** Hetron M. Munang’andu

**Affiliations:** Section of Aquatic Medicine and Nutrition, Department of Basic Sciences and Aquatic Medicine, Faculty of Veterinary Medicine and Biosciences, Norwegian University of Life SciencesOslo, Norway

**Keywords:** viral metagenomics, environment, aquaculture, epidemiology, aquatic, organisms

## Abstract

Studies on the epidemiology of viral diseases in aquaculture have for a long time depended on isolation of viruses from infected aquatic organisms. The role of aquatic environments in the epidemiology of viral diseases in aquaculture has not been extensively expounded mainly because of the lack of appropriate tools for environmental studies on aquatic viruses. However, the upcoming of metagenomics analyses opens great avenues in which environmental samples can be used to study the epidemiology of viral diseases outside their host species. Hence, in this review I have shown that epidemiological factors that influence the composition of viruses in different aquatic environments include ecological factors, anthropogenic activities and stocking densities of cultured organisms based on environmental metagenomics studies carried out this far. Ballast water transportation and global trade of aquatic organisms are the most common virus dispersal process identified this far. In terms of disease control for outdoor aquaculture systems, baseline data on viruses found in different environments intended for aquaculture use can be obtained to enable the design of effective disease control strategies. And as such, high-risk areas having a high specter of pathogenic viruses can be identified as an early warning system. As for the control of viral diseases for indoor recirculation aquaculture systems (RAS), the most effective disinfection methods able to eliminate pathogenic viruses from water used in RAS can be identified. Overall, the synopsis I have put forth in this review shows that environmental samples can be used to study the epidemiology of viral diseases in aquaculture using viral metagenomics analysis as an overture for the design of rational disease control strategies.

## Introduction

Viruses are the most abundant biological agents on the planet with the majority being found in the freshwater and marine water environments (Fuhrman, 1999; Edwards and Rohwer, 2005). They infect all living organisms including prokaryotes and eukaryotes. They are a major cause of mortality for living organisms and they are a reservoir of the greatest genetic diversity on Earth (Fuhrman, 1999; Edwards and Rohwer, 2005). Viruses can be moved between marine and terrestrial reservoirs by different dispersal mechanisms thereby raising the specter of emerging diseases (Suttle, 2005). As pointed out by Edwards and Rohwer (2005), identifying and monitoring the dynamics of environmental viral communities is a complex challenge because it is estimated that only <1.0% of environmental viruses have been cultivated and characterized this far while the majority are unknown. Although Breitbart et al. (2002) have shown that it is possible to sequence entire genomes of uncultured marine viruses using metagenomics, Kim et al. (2013) pointed out that taxonomic profiling of microbial communities using operational taxonomic units (OTU) used to identify the most dominant and rare OTUs in environmental samples is still under development as most databases do not reach the full extent of microbial diversity classification. Unlike bacteria that use the 16S ribosomal RNA as a common gene for their identification, viruses lack a single common gene for their identification which makes it difficult to monitor their population dynamics in different aquatic environments. Despite so, the current ongoing global expansion of aquaculture, which is solely dependent on freshwater and marine environments for the culture of aquatic organisms demands for a better understanding of epidemiological factors that influence the composition of viruses found in aquatic environments in order to pave way for the rational design of effective disease control strategies (Edwards and Rohwer, 2005).

One of the prime objectives for carrying out viral epidemiological surveys in the environment is to evaluate the taxonomic composition of viruses found in different ecosystems (Lindner and Renard, 2013). However, our knowledge on the epidemiology of viral diseases in aquaculture is to a large extent derived from virus isolation studies carried out on infected aquatic organisms. While this approach has significantly increased our epidemiological knowledge of viral diseases in aquaculture, it lacks detail on the role of environmental factors on the epidemiology of viral diseases. This is mainly because there has been a general lack of appropriate tools able to identify and characterize viruses collected from environmental samples. However, the emergence of high throughput next generation sequencing (NSG) in recent years has not only enhanced our understanding of host responses to viral infections (Chu et al., 2015; Dahle et al., 2015; Xu et al., 2015, 2016a,b; Liu et al., 2016), but it has opened a great avenue for identification of viruses found in different aquatic environments using metagenomics analyses (Suttle, 2005, 2007; Djikeng et al., 2009; Kim et al., 2015). Viral metagenomics is a culture independent sequencing tool able to identify a large number of viral genomes from the same sample at the same time without prior knowledge of genomic sequences of the viruses to be identified (Handelsman, 2004; Bibby, 2013). Since its discovery, it has been used for the identification of viruses in water samples collected from different ecosystems (Suttle, 2005, 2007; Djikeng et al., 2009; Kim et al., 2015), which creates the basis for studying viral pathogens from environmental samples outside their hosts. However, it is noteworthy that metagenomics alone is inadequate to provide a comprehensive understanding of factors that influence microbial composition in freshwater and marine environments given the vastness of data generated by this tool, which makes it difficult to discern important patterns and discriminators of microbial compositions in environmental samples (Dinsdale et al., 2013). And as such, a wide range of clustering, classification, and visualization tools such as the multivariate analysis and taxonomic clustering using OTU based approaches have been developed to complement the analyses of metagenomics data (Dinsdale et al., 2008a,b, 2013; Edgar, 2010; Cai and Sun, 2011; Fu et al., 2012). Nevertheless, a good understanding of environmental factors that influence the epidemiology of viral diseases in aquaculture using these tools is cardinal for the rational design of effective disease control strategies.

Hence, this review brings into perspective the use of viral metagenomics analyses in identifying environmental factors that influence the epidemiology viral diseases in aquaculture. It explores the use of viral metagenomics analyses in identifying environmental factors that influence the diversity and composition of viral communities found in different environments. It also brings into perspective the different viral dispersal mechanisms identified this far and highlights the factors that influence the persistence of viruses in different aquatic environments. Finally, it also provides insight on the potential use of viral metagenomics analyses in the design of effective disease control strategies in aquaculture.

## Factors Influencing the Composition Viral Communities in Freshwater Environments

This far, freshwater metagenomics surveys have been carried out in ponds (Rodriguez-Brito et al., 2006; Dinsdale et al., 2008a), hydrocarbon polluted water (Abbai et al., 2012), reclaimed water (Rosario et al., 2009), man-made recreational lakes (Bench et al., 2007), and natural freshwater lakes (Roux et al., 2012). Based on observations from these studies, some of the factors influencing the composition of viral communities in freshwater environments include anthropogenic factors, seasonal variations, recycled water management systems, and ecological factors as shown below.

### Anthropogenic Factors

Several studies have shown that human activities have a significant influence on the diversity and composition of microbial organisms in different freshwater ecosystems (Horner-Devine et al., 2004; Djikeng et al., 2009; Fancello et al., 2013). For example, Fancello et al. (2013) showed that viral communities found in the Mauritanian Sahara desert ponds were partly influenced by agriculture activities from surrounding areas. The viral genomes detected in some of the ponds included the *Spodoptera litura* nuclear polyhedrosis virus (NPV), which is a baculovirus that infects dates producing plants. Djikeng et al. (2009) identified several terrestrial viruses in Lake Needwood in the USA with possible agriculture and public health implications. They identified several viruses originating from farmed plants, animals, birds, and fish from water samples collected from Lake Needwood. They also detected Banna virus, which is a mosquito borne zoonotic virus mainly found in the tropical climates of South East Asia. As for aquatic viruses, Djikeng et al. (2009) detected several fish viruses that included Atlantic salmon nervous necrosis virus (ASNNV), Atlantic halibut nodavirus (AHNV), red spotted grouper nervous necrosis virus (RSGNNV), and striped jack nervous necrosis virus (SJNNV) which are pathogens of farmed fish. They also detected Taura syndrome virus (TSV) and white spot syndrome virus (WSSV), which are pathogens of farmed shrimps. These findings suggest that human involvement could have played a role in introducing fish and shrimp viruses into Lake Needwood. Overall, these observations show that freshwater viral communities include pathogens of terrestrial and aquatic organisms. Therefore, it is likely that anthropogenic activities that alter the composition of terrestrial and aquatic host organisms have a significant influence on the composition of the viruses found in freshwater environments.

Kim et al. (2015) carried a viral metagenomics surveillance of the Ballast water discharged by ships on the shores of the North American Great Lakes. Ballast water is water carried in ballast tanks of large marine vessels such as ship cruises, tankers, and large cargo carriers in order to improve their stability and balance. As pointed out by Hayes and Sliwa (2003), a wide variety of organisms spread by ballast waters may establish themselves in new environments when discharged from ships, which could alter the receiving ecosystems. Kim et al. (2015) collected ballast water samples from five bulk ship carriers arriving at different Ports in the Great Lakes in which they detected several shrimp viruses that included WSSV, TSV, and infectious myonecrosis virus (IMNV) using viral metagenomics. They also detected fish viruses that included strip-jack nervous necrosis (SJNNV), Cyprinid herpesvirus 3 (CyHV-3), infectious spleen and kidney necrosis virus (ISKNV), and hemorrhagic septicemia virus (VHSV). These findings show that ballast water transportation could play a vital role in the dispersal viruses into freshwater environment.

### Recycled Water Management

Rosario et al. (2009) surveyed the virome of reclaimed wastewater from different counties in Florida. The metagenome unraveled a wide range of animal, human, invertebrate, and plant viruses found in reclaimed water including viruses that resist disinfection after wastewater treatment. In their study, they observed that reclaimed water offers a great opportunity for testing the efficacy of different disinfection methods used for treating recycled water. Hence, data from these studies can be used to optimize disinfection procedures used to eliminate pathogenic viruses from recycled water. In aquaculture, this approach can be used to identify pathogens that resist disinfection in recirculation aquaculture systems (RAS). Unlike the outdoor culturing of aquatic animals in open ponds, lakes or sea cages, RAS cultures aquatic animals at high densities using indoor facilities in a controlled water recirculation environment. RAS has been used for the culture of different aquatic organisms such as shrimps (Lin et al., 2003), tilapia (Rafiee and Saad, 2005), seabass (Franco-Nava et al., 2004), catfish (Miller and Libey, 1984), and cobia (Resley et al., 2006). Water used in RAS is filtered and disinfected using different methods such as ultraviolent (UV) light exposure or ozone treatment before it is recycled back into culture tanks. However, it is not known whether all these disinfection procedures are effective at inactivating all floating viruses in RAS. Therefore, viral metagenomics analysis can be used to identify viruses that resist disinfection in RAS and help optimize the efficacy of disinfection procedures for the elimination of pathogenic viruses before the water is recycled back in the culture tanks.

### Ecological Factors of Freshwater Ecosystems

The major ecological factors influencing the composition of viral communities in freshwater environments include host species composition and abundance as well as factors that influence seasonal variations as shown below.

#### Host Species Composition and Abundance

The major factors shown to influence the viromes in freshwater ecosystems include the host species composition, diversity, and abundances of the potential hosts, which has been shown to vary with the trophic status, depth, watershed, or size (Lefranc et al., 2005; Boucher et al., 2006; Roux et al., 2012). Roux et al. (2012) pointed out that viral community diversity correlates with the diversity of organisms seen to be potential hosts. Roux et al. (2012) compared the viral metagenomes of two lakes located in two separate continents and showed that freshwater clades were similar between lakes in different continents. Phylogenetic analysis of major groups show that two viral communities were closely related despite significant ecological differences between the two lakes suggesting that the two lakes were composed of evolutionary close virotypes but only differed in terms of the relative abundance of viral species. They pointed out that virus communities appeared to cluster hierarchically according to salinity levels. In terms of aquaculture, these findings suggest that viruses infecting freshwater aquatic organisms are bound to be more prevalence in the Lakes, ponds and rivers than in marine environments. This is supported by observations made by Roux et al. (2012) who showed that viruses from freshwater environments cluster together despite the vast geographical distances between sample locations. Moreover, this is also in line with observation made from two independent studies carried out by Kim et al. (2015) who reported SJNNV, WSSV, and TSV in the Great Lakes and Djikeng et al. (2009) who detected similar viruses in Lake Needwood and yet the two areas are several kilometers apart in USA and they do not share a common water-flow. However, there is need for more detailed studies to consolidate this notion given that viruses such as viral hemorrhagic septicemia virus (VHSV) have been detected from both freshwater and marine fish species (Brudeseth et al., 2008). **Table 1** shows some of the pathogenic viruses for fish and crustaceans used in aquacultures detected from different freshwater environments by viral metagenomics analysis.

**Table 1:** Finfish and crustacean viruses detected by metagenomics in freshwater environments.

Virus	Abbreviation	Family	Genome	Reference
White spot syndrome virus	WSSV	Nimaviridae	dsDNA	Suttle, 2005; Kim et al., 2015
Taura syndrome disease virus	TSV	Dicistroviridae	ssRNA	Djikeng et al., 2009; Rosario et al., 2009; Kim et al., 2015
Lymphocytic disease virus	LCDV	Iridoviridae	dsDNA	Rosario et al., 2009
Rockbream iridovirus	RBIV	Iridoviridae	dsDNA	Rosario et al., 2009
Atlantic cod nervous necrosis virus	ACNNV	Nodaviridae	ssRNA	Djikeng et al., 2009
Atlantic halibut noda virus	AHNV	Nodaviridae	ssRNA	Djikeng et al., 2009
Red spotted grouper necrosis virus	RSGNV	Nodaviridae	ssRNA	Djikeng et al., 2009
Strip-jack nervous necrosis virus	SJNNV	Nodaviridae	ssRNA	Djikeng et al., 2009; Kim et al., 2015
Infectious spleen and kidney necrosis	ISKNV	Iridoviridae	dsDNA	Kim et al., 2015
Cyprinid herpesvirus 3 (koi herpes virus)	CyHV-3	Herpesviridae	dsDNA	Kim et al., 2015
Shrimp infectious myonecrosis virus	IMNV	Totiviridae	ssRNA	Kim et al., 2015
Viral hemorrhagic septicemia virus	VHSV	Rhabdoviridae	ssRNA	Kim et al., 2015

#### Seasonal Variations

Tseng et al. (2013) found a positive correlation between changes in precipitation and viral composition in different freshwater environments. In the 2-year survey carried out in an enclosed freshwater reservoir in Feitsui, Taiwan, subjected to episodes of typhoons, Tseng et al. (2013) showed that the intermittent addition of terrestrial viruses contributed to the increase in the diversity of viral communities. They noted that viral abundance followed seasonal variations, increasing in summer, and decreasing in winter. They attributed the high viral diversity in summer to the higher host abundance and activity unlike in winter when the host abundance and activity was low. Similarly, Djikeng et al. (2009) showed that the overall taxonomic distribution of RNA viruses detected in Lake Needwood showed a higher viral diversity in samples collected in June than in November. They observed that some viruses such as influenza. A virus were only detected in winter while Banna virus, which is a mosquito-borne virus mostly reported in tropical climates, together with avian encephalomyelitis were largely detected in summer. As pointed out by Tseng et al. (2013), it is not clear as to whether some of the terrestrial viruses found in freshwater ecosystems can be infectious to aquatic organisms or they are simply a transient passing signal of freshwater input. To better understand this phenomenon, there is need for detailed studies to elucidate the role of these viruses in freshwater ecosystems and their impact on aquaculture (Tseng et al., 2013). However, as pointed out by Sobsey and Pillai (2009) that some of waterborne microbes currently found in freshwater and marine environments could emerge as future novel pathogens that will cause disease in different host species as a result of adaptation and evolutionary changes involving a variety of abiotic and biotic factors. Hence, there is need for continuous epidemiological surveys in order to elucidate the possibility of some terrestrial viruses evolving into pathogens of aquatic organisms.

Different scientists have shown that the distribution and composition of viruses changes with extreme climatic changes such as heavy rainfall, flooding, and typhoons (Hewson et al., 2012; Tseng et al., 2013). For example, Nakayama et al. (2007) showed that viral abundance in floodwater of Japanese paddy field was larger than in natural marine and freshwater environments while Tseng et al. (2013) showed increased microbial richness after several typhoon events on the Feitsui Reservoir in Taiwan. Overall, these studies show that the relative abundance and diversity of viral communities changes with seasonal variations and extreme climatic changes in freshwater ecosystems.

## Factors that Influence the Composition of Viral Communities in Marine Environments

This far, viral metagenomics studies have covered almost all the major oceans to include the Arctic (Hingamp et al., 2013; Brum et al., 2015; Sunagawa et al., 2015), Antarctic (Suttle, 2005; López-Bueno et al., 2009), sub-Antarctic (Kluge et al., 2016) pacific (Hurwitz and Sullivan, 2013), Indian (Williamson et al., 2012), and Atlantic oceans (Suttle, 2005; Tucker et al., 2011). Based on observations from these studies, the most common environmental factor shown to influence the compositions of viruses in coastal areas used for aquaculture are anthropogenic activities and ecological factors.

### Anthropogenic Factors

Anthropogenic factors shown to influence the composition of viral communities in different in marine environments used for aquaculture include ballast water transportation, industrial activities and the culture of aquatic animals as shown below.

#### Industrial and Recreational Activities

Several human-derived activities such as pollution, coastal engineering, urban development, oil extraction, refinery, mining, and agriculture have been shown to alter the microbial composition of different marine ecosystems (Islam and Tanaka, 2004; Halpern et al., 2008; Nogales et al., 2011). Dinsdale et al. (2008b), showed that microbial communities from coral atolls in the Pacific ocean drastically changed along a gradient of human disturbance with the most anthropogenic impacted atoll being dominated with microbes that included human pathogens. The extents to which these activities affect the composition of viruses that infect aquatic animals in coastal areas vary depending on the industrial activity being carried out. Moreover, as coastal communities continue to expand alongside the increase in the culture of aquatic animals in marine environments, the specter of public health risks also continues to increase which includes exposure of humans to zoonotic viruses. Viruses reported to infect humans in marine environments include Marine calicivirus, which has been isolated from the San Miguel Sea lions (*Zalophus californianus*), amphibians, fish, and cetaceans (Smith et al., 1973, 1980; Smith and Boyt, 1990). This virus has been show to survive up to 15 days in marine water (Smith et al., 1981). Seal poxvirus causes cutaneous lesions resembling other zoonotic parapoxvirus infections such as Orf virus in humans (Hicks and Worthy, 1987; Clark et al., 2005) while rotavirus group C which causes diarrhea in humans was recently isolated from the Antarctica sea fur using viral metagenomics (Kluge et al., 2016). Given that water is a stable medium that allows for the transmission of viruses, the shedding of zoonotic viruses in marine environments could serve as a mode of transmission to humans. However, to fully understand the specter of emerging viral zoonosis and their mode of transmission in marine environments there is need for extensive environmental surveillance studies.

#### Ballast Water Transportation

Ballast water transportation has increased tremendously due to an upsurge in maritime traffic in recent decades with most water discharges being done in coastal areas near aquaculture facilities. Shipping accounts for two thirds of the world trade in metric tons (Hoffmann and Kumar, 2010). By 2004, global ballast water volume discharged into open sea was estimated at 2800 million tons (Endresen et al., 2004). It is estimated that ocean vessels transport up to 12 billion tons of ballast water each year serving as mechanism of transferring aquatic life from one part of the world to the other (Tamelander et al., 2010). Kim et al. (2016) isolated Penaeid shrimp infectious myonecrosis virus (PsIMNV) from Singapore harbor water and ballast water in Western Asia, South East Asia and Pacific Ocean. As mentioned by Walker and Winton (2010), PsIMNV has undergone a wide global distribution in aquaculture starting from Brazil where it was first reported, spreading to Indonesia, Thailand, and Hainan Province in China. Its detection in ballast water in Los Angeles in North America where the disease has not been reported demonstrates the potential role of ballast water in the spread viral disease in aquaculture (Kim et al., 2016). Similarly, red sea bream iridovirus (RISV) was detected in ballast water discharged in the Los Angeles harbor in North America by viral metagenomics surveys (Kim et al., 2016) and yet outbreaks of RSIV have mostly been reported in Asia (Jung and Oh, 2000; Wang et al., 2002; Kim et al., 2005; Imajoh et al., 2007; Jeong et al., 2008; Shinmoto et al., 2009) further demonstrating that ballast water plays a vital role in the spread of viral diseases in aquaculture. In addition, these findings show that environmental metagenomics surveillance can serve as an early warning system able to detect pathogens in areas where the disease has not been previously reported.

#### Culture of Aquatic Organisms

As mentioned by Walker and Winton (2010), anthropogenic activities in aquaculture have led to displacement of aquatic animals from their natural environments to highly overstocked densities in confined habitats where they are given artificial feed to enhance production. Epidemiological models predict that viral infection rates increase with higher host stocking density given that infection is a direct function of contact between the host and pathogen (Wiggins and Alexander, 1985; Murray and Jackson, 1992; Suttle and Chan, 1993; Wilcox and Fuhrman, 1994). Hence, the higher the stocking densities of susceptible host populations to viral infections, the higher the chances of changing the viral community in that environment. Therefore, the anthropogenic stress and overstocking impacted on cultured animals does not only serve as a mode of virus amplification by causing high mortalities that lead to shedding of high viral quantities into the environment, but it also serves as a mode of changing the viral composition in marine environments.

In addition to intensified culture systems, there has been a prolific global increase in the trade of aquatic animals (Walker and Winton, 2010), which has significantly contributed to the spread of viral diseases across the world. For example, infectious hematopoietic necrosis virus (IHNV) was first reported in the USA as a disease of cultured rainbow trout. It spread to Asia through contaminated eggs where it causes disease in rainbow trout as an exotic species introduced from the USA (Walker and Winton, 2010). The spread of Koi herpesvirus (KHV) has been linked to the trade of persistently infected fish that release the virus in new environment (Haenen et al., 2004). Similarly, the rapid spread of TSV and WSSV in the 1990s was attributed the increase in the trade of live and frozen shrimps (Lightner et al., 1997). To better understand the impact of these anthropogenic activities on the diversity and composition of viromes found in marine environments used for aquaculture, there is need for extensive metagenomics surveillance studies. Such studies would help elucidate the changes in the viral composition induced by introducing exotic host species in marine environments. Further, such studies would shed insight on the impact of high stocking densities on viral quantities released in the environment, which would help determine the optimal environmental concentration of pathogenic viruses required to induce outbreaks in susceptible host populations. As a consequence, these studies would help design effective disease control strategies required to prevent disease transmission by monitoring the environmental quantities of floating viruses.

### Ecological Factors

Ecological factors that influence marine viromes have been extensively reviewed by different scientists (Wommack and Colwell, 2000; Suttle, 2005, 2007; Delwart, 2007; Wawrzynczak, 2007; Dinsdale et al., 2008a, 2013; Mokili et al., 2012; Brum et al., 2015). Although there are several factors likely to influence the epidemiology of viral diseases in aquaculture, some of the prominent factors identified this far include the biogeographical, abiotic and biotic factors as shown below.

#### Biogeographic Factors

Marine studies carried out this far show that the diversity and composition of viral communities vary with differences in biogeographical location. Angly et al. (2006) sampled over 184 sites from 64 different places located in Sargasso Sea, Arctic Ocean, Gulf of Mexico, and coastal area of British Colombia. They showed that the richness of viral species obtained from these areas varied along a latitudinal gradient and that diversity was highest in regions nearest to the equator and lower in areas toward the poles. In addition, they observed that marine viral communities from British Colombia, which is an area affected by seasonal upsurge and outflow of many rivers was exceptionally genotype-rich as a result of the inflow from other regions. Dinsdale et al. (2008b), analyzed 32 viromes from four biogeographic regions based on different parameters that included seasonal variations, ocean depth, and proximity to land. In their findings, they observed that viral richness decreased from deep sea to surface waters and with distance from shore in surface waters. They also observed that viral richness increased from winter to summer. These findings show that coastal areas, which are the most ideal sites for aquaculture use tend to have the highest viral richness, which could raise the specter of emerging pathogens in aquaculture. Therefore, these findings indicate that potentially high-risk areas able to harbor pathogenic viruses can be identified using viral metagenomics surveys. And as such, metagenomics surveillance can be used to select aquaculture sites that are less endemic to viral pathogens for the culture of aquatic animals in marine environments.

#### Abiotic Factors

Different viral metagenomics studies have shown that marine viral communities are influenced by different abiotic factors such as temperature, salinity, and oxygen concentrations (Kukkaro and Bamford, 2009; Brum et al., 2015). Cassman et al. (2012) showed that reduction in oxygen levels corresponded with the shedding of viruses from infected eukaryotic organisms. Brum et al. (2015) showed that variable inherent decay rates of different viruses could affect their persistence or degradation in nature implying that viruses with lower decay rate will persist longer in the environment while those with higher decay rate will degrade quickly (Wommack and Colwell, 2000). Decay rates tend to follow the overall biological richness of the waters, with the fastest decay occurring in rich nearshore waters while slowest decays occur in low nutrient offshore water (Brum et al., 2015). Overall, these observations show that different ecological factors influence the survival and persistence of different viruses in different marine environments.

#### Biotic Factors

Several metagenomics studies have shown that viral community structures follow the environmental conditions shaping the host community (Fuhrman, 1999; Matteson et al., 2012; Sunagawa et al., 2015) given that viruses require host organisms for their replications. For example, Matteson et al. (2012) showed an increase in the production of viral particles for viruses infecting bacteria during a spring phytoplankton boom in the South Pacific Ocean in New Zealand in which the production rate of viral particles increased corresponding with bacteria mortality while Ory et al. (2011) showed that the high peaks of phytoplankton during the autumn period of 2007 may have led to increase in the population of viruses that specifically infect phytoplankton as shown that the viral fingerprint pattern generated uniquely correlated with the occurrence of phytoplankton viruses. In aquaculture this implies that the type of aquatic organisms cultured in a given ecosystem shapes the viral community in that ecosystem. Hence, marine environments engaged in shrimp farming promote the increase of viruses that infect shrimps while places engaged in fish farming promote the increase of viruses that infect fish species. This phenomenon implies that changes in the host community brought about by introducing exotic species or selective culture of host species used for human consumption, ornamental utilization, or recreational activities plays a pivotal role in altering the diversity and composition of viruses found in the places used for aquaculture. And as pointed out by Sobsey and Pillai (2009), that some currently existing waterborne microbes could become future emerging pathogens arising from evolutionary or adaptation changes caused by a variety of abiotic and biotic mechanisms. There is evidence of both adaptive and evolutionary changes in virus and host receptors, which can render non-virulent viral strains to become pathogenic (Baranowski et al., 2001; Rossmann et al., 2002). For example, in our studies we have shown that a few amino acids strategically located on the outer surface of the infectious pancreatic necrosis virus (IPNV) capsid account for virulence and immunogenicity properties of IPNV in Atlantic salmon (Song et al., 2005; Munang’andu et al., 2013; Mutoloki et al., 2013, 2016). We have shown that non-pathogenic strains can revert to virulence based on mutations of two amino acid substitutions on the surface capsid of IPNV rendering avirulent strains to become virulent when fish infected with none pathogenic strains are subjected to stress (Gadan et al., 2013). Hence, it is likely that other viruses could use similar mechanisms to evolve from avirulent to virulent strains in different aquatic organisms.

## Major Drawbacks Limiting the Use of Viral Metagenomics

Although metagenomics has proved to be a powerful tool able to sequence all nucleic acids present in a sample leading to identification of viral pathogens without prior knowledge of their genomic sequences, it has significant limitations that require the support of other tools to enhance its application. The vastness of the data generated requires long computation time and needs the support of other tools for clustering, classification and identification of individual viral sequences. Prakash and Taylor (2012) pointed out that the read length generated during sequencing has a strong influence on assembly, gene prediction, and the ultimate sequence analysis. They noted that the most reliable functional annotation was by homology based approaches against publicly available reference sequences. The major drawback for this approach is that functional annotation has generally lagged behind the rate at which metagenomics data is being generated. Moreover, the number of publicly available annotation databases for aquatic viruses is limited (Edwards and Rohwer, 2005). Further, homology based approaches take a long time to identify homologs of each sequence in the metagenome. And as such, alternative methods have been developed such as phylogenetic profiling (Pellegrini et al., 1999) and neighborhood tree alignments (Dandekar et al., 1998; Overbeek et al., 1999). The major limitation with these approaches is that in the absence of background information of infected host species that shed viruses in the environment it is difficult to link viral genomes from environmental samples with disease infections, which makes it difficult to identify novel pathogens from environmental samples.

For metagenomic sequences linked to novel diseases, there is need to isolate the virus involved followed by verification using conventional diagnostic approaches such as cell culture to exhibit the cytopathic effect (CPE), morphological characterization using electron microscopy, and molecular characterization using PCR (Bibby, 2013). In addition, it is important to demonstrate the ability of the isolated virus to cause pathognomonic features of the disease in susceptible hosts. For unculturable viruses, verification can be done by PCR although Yang et al. (2011) showed a lack of agreement between metagenomics and PCR results in which they attributed the lack of correlation between the two techniques to different factors such as sequencing biases, errors in assembly or annotation and possibility of false PCR results. Overall, these factors show that metagenomics is primarily a tool used for sequencing all nucleic acids present in a sample, which requires the support of other tools to demonstrate the ability of the viruses identified to cause disease in susceptible host species.

## Conclusion

This review has shown that viral metagenomics can be used to study the epidemiology of viral diseases in aquaculture using environmental samples. Epidemiological factors that influence the composition of viruses in aquatic environments include ecological factors, anthropogenic activities, introduction of exotic species and high stocking densities of cultured animals. Ballast water transportation and global trade of aquatic animals are the most common virus dispersal methods identified this far. As for disease control, environmental samples can be used to obtain baseline data of pathogenic viruses found in different aquatic environments. And as such, high-risk areas having a high specter of pathogenic viruses can be identified as an early warning system. As for indoor RAS, viral metagenomics analyses can be used to identify the most effective disinfection methods able to eliminate pathogenic viruses from recirculation water before it is put back in culture tanks. Overall, the synopsis I have put forth in this review shows that the upcoming era of viral metagenomics has opened great avenues in aquaculture virology enabling us to study the epidemiology of viral diseases using environmental samples as an overture to develop effective disease control strategies.

## Author Contributions

The author carried out the literature review and preparation of the manuscript.

## Conflict of Interest Statement

The author declares that the research was conducted in the absence of any commercial or financial relationships that could be construed as a potential conflict of interest.
